# Country-Level Cost-Effectiveness Thresholds: Initial Estimates and the Need for Further Research

**DOI:** 10.1016/j.jval.2016.02.017

**Published:** 2016-12

**Authors:** Beth Woods, Paul Revill, Mark Sculpher, Karl Claxton

**Affiliations:** Centre for Health Economics, University of York, Heslington, York, North Yorkshire, UK

**Keywords:** benefits package, cost-effectiveness, quality-adjusted life-years, threshold, universal health care, willingness to pay

## Abstract

**Background:**

Cost-effectiveness analysis can guide policymakers in resource allocation decisions. It assesses whether the health gains offered by an intervention are large enough relative to any additional costs to warrant adoption. When there are constraints on the health care system’s budget or ability to increase expenditures, additional costs imposed by interventions have an “opportunity cost” in terms of the health foregone because other interventions cannot be provided. Cost-effectiveness thresholds (CETs) are typically used to assess whether an intervention is worthwhile and should reflect health opportunity cost. Nevertheless, CETs used by some decision makers—such as the World Health Organization that suggested CETs of 1 to 3 times the gross domestic product (GDP) per capita—do not.

**Objectives:**

To estimate CETs based on opportunity cost for a wide range of countries.

**Methods:**

We estimated CETs based on recent empirical estimates of opportunity cost (from the English National Health Service), estimates of the relationship between country GDP per capita and the value of a statistical life, and a series of explicit assumptions.

**Results:**

CETs for Malawi (the country with the lowest income in the world), Cambodia (with borderline low/low-middle income), El Salvador (with borderline low-middle/upper-middle income), and Kazakhstan (with borderline high-middle/high income) were estimated to be $3 to $116 (1%–51% GDP per capita), $44 to $518 (4%–51%), $422 to $1967 (11%–51%), and $4485 to $8018 (32%–59%), respectively.

**Conclusions:**

To date, opportunity-cost-based CETs for low-/middle-income countries have not been available. Although uncertainty exists in the underlying assumptions, these estimates can provide a useful input to inform resource allocation decisions and suggest that routinely used CETs have been too high.

## Introduction

Policymakers in all health care systems face difficult choices about which interventions, programs, or activities (hereinafter referred to solely as “interventions”) should be funded from limited available resources. The tools of economic evaluation offer various means to assist policymakers in the process of prioritization. A common approach is the incremental cost-effectiveness analysis (CEA), which is based on the comparative assessment of costs and benefits, with the latter generally focused on health gains. CEA seeks to identify which interventions offer health gains large enough, relative to their costs, to warrant adoption [1].

CEA typically includes detailed information about the incremental costs (∆costs) and the incremental health effects (∆health) of an intervention relative to alternative interventions. The results of CEA are often expressed as an incremental cost-effectiveness ratio (ICER), the ratio of incremental costs to incremental health effects (∆costs/∆health) [1]. Health effects are often represented as quality-adjusted life-years (QALYs) gained or disability-adjusted life-years (DALYs) averted, and so the ICER gives the “cost per QALY gained/DALY averted” associated with an intervention. Although these are useful summaries, the question remains as to whether a particular cost per QALY gained/DALY averted ought to lead to the evaluated intervention being considered cost-effective.

If an intervention offers incremental health gains but at some additional costs, then a decision regarding whether it should be funded should be informed by the value of what will be given up as a consequence of those costs (i.e., the opportunity cost of funding the intervention [2]). All systems face some restrictions on the resources available for health care. If resources are committed to the funding of one intervention, then they are not available to fund and deliver others. The opportunity cost of a commitment of resources is, therefore, the health forgone because these “other” interventions that are available to the health system cannot be delivered. Even if additional resources are placed into the health care system to be made available for a particular new intervention, there is an opportunity cost to these resources—the health that could have been gained by investing these additional resources elsewhere in the system.

In the context of CEA, the opportunity cost can be expressed using a cost-effectiveness threshold (CET). CETs based on opportunity costs describe the amount of money that, if removed from the health care system, would result in one less unit of health being generated, or equivalently, the cost of generating health in the present system. In the case of the introduction of a new intervention that imposes additional costs on the system, this is equivalent to a marginal reduction in the resources available for other activities. If the ICER (cost per QALY gained/DALY averted) is less than the CET, it means that diverting funds to the intervention will increase population health. For example, if the CET is $1000/QALY and the ICER for an intervention is $100/QALY, then for every $1000 spent on the intervention 1 QALY is lost in the wider health care system but 10 are gained from the new intervention. The net health effect is positive. Therefore, if an ICER is less than the CET, an intervention can be considered cost-effective, but if an ICER is more than the CET, the benefits are insufficient in comparison with costs and the intervention cannot be considered to be cost-effective. Hence, CEA simplifies to an assessment of whether a new intervention will result in gains in population health and the inverse of the CET should reflect the marginal product of health care spending (∆health/∆costs).

Estimating the opportunity cost of health care spending (i.e., estimating the CET) is, therefore, a crucial aspect of any resource allocation decision in health care.

### Understanding CETs

Recent methods research has emphasized the centrality of opportunity costs in informing resource allocation decisions and how CETs can be appropriately estimated for CEA to inform decisions aimed at improving population health [3], [4] (see (Chapter 4 of Drummond et al. [1] for a full overview). A clear distinction needs to be made between two related, but separate, concepts that have informed the debate regarding the most appropriate value for the CET: 1) opportunity costs in terms of health foregone when costs fall on health care budgets and 2) opportunity costs in terms of foregone consumption (the “consumption value of health”) when additional costs fall on consumption opportunities outside health care. The first is an issue of “fact,” resulting from limits in the overall collective budget available for health care or constraints on the health system’s abilities to increase expenditure. It reflects the health generated at present from the health care system (or that could be gained if expenditure were increased) and, therefore, reflects the “supply side” of the system. The second is an issue of “value” and depends on how individuals and society value health as compared with other forms of consumption or publicly funded nonhealth goods. This indicates what individuals and society want from the health care system, or the “demand side.”

For economic evaluation it is important to consider what type of opportunity costs would result from investment in new activities. If opportunity costs result in the form of health forgone (e.g., through displacement of other health-generating interventions), then the CET should reflect this (let’s denote this as “*k*,” the amount of money that would displace one QALY’s worth of health care investment). If opportunity costs are in terms of other forms of consumption, then the CET should reflect the consumption value of health (let’s denote this as “*v*”).

If we observe that the consumption value of health is higher than the amount of health care resource required to improve health (i.e., if *v* > *k*), then this suggests that the health care system is not meeting individual preferences. Individuals would be willing to give up more of the resources available to them to improve their own health than the health care system would require. There are a number of reasons why this may be the case, not least the welfare losses associated with socially acceptable ways to finance health care systems and the fact that individuals may be willing to expend more resources in improving their own health than in improving the health of others via a collectively funded system.

For incremental CEA to inform the allocation of health care expenditures, for which the primary purpose is generally regarded as being the generation of health from limited collective health care resources, CETs reflecting the opportunity costs of health care spending (*k*) will always be required if there are any restrictions on the growth in health care expenditure (see Chapter 4 of Drummond et al. [1]).

### Estimating Cost-Effectiveness Thresholds

CETs have not generally been set to reflect *k*. For instance, values of £20,000 to £30,000 and $50,000 have commonly been applied in the United Kingdom and the United States, respectively [5], [6]. Similarly, for low- and middle-income countries, the World Health Organization (WHO) has recommended thresholds of 1 to 3 times the gross domestic product (GDP) per capita [7]. These values are not based on assessment of health opportunity costs resulting from resource constraints. The basis for these thresholds is unclear; they, however, appear to have been conceptually and to some degree empirically informed by the consumption value of health (or more accurately, estimates of individuals’ willingness to pay [WTP] to improve their own health). For instance, the WHO threshold is described as being based on estimates reported in the Commission on Macroeconomics and Health report from 2001 [8]. These estimates were intended to inform decisions regarding overall investments in health care spending and used estimates of the WTP for mortality risk reductions. Indeed, similar approaches continue to be used to advocate for increased health care spending [9]. Nevertheless, the use of these thresholds when assessing the value of individual interventions in the context of existing spending limits is not consistent with population health improvement, because they do not reflect the opportunity costs that are imposed on health care systems. Although demand-side thresholds might inform social choices about the magnitude of financial resources committed to health care, they are inappropriate measures of health opportunity cost and so risk reducing, rather than increasing, population health when used in the context of CEA.

Alternatively, the relationship between changes in health care expenditure and health outcomes—the marginal productivity of the health care system in generating health—can be estimated. This provides a direct measure of the health consequence of changes in available resources, for example, when a cost-escalating intervention is adopted or what could be gained if additional resources are made available in general to fund health care. Using such estimates of *k* to inform CETs provides a basis for informing resource allocation decisions with a view to increasing population health. There is, however, a paucity of estimates of CETs using these approaches. One notable exception is in the study by Claxton et al. [4] which used local-level program expenditure data, in a range of disease areas, to estimate the relationship between changes in health care expenditure and health outcomes in the English National Health Service (NHS) (see Chapter 4 of Drummond et al. [1] for a full description of this work).

By exploiting the variation in expenditure and in mortality outcomes, Claxton et al. estimated the relationship between changes in spending and mortality in those clinical program areas in which a mortality effect could be identified while accounting for endogeneity. With additional information about age and sex of the patient population, these mortality effects were expressed as a cost per life-year threshold (£25,241 per life-year). These life-year effects were adjusted for quality of life using additional information about quality-of-life norms by age and sex as well as the quality-of-life impacts of different types of disease. By using the effect of expenditure on the mortality and life-year burden of disease as a surrogate for the effects on a more complete measure of health burden (i.e., which includes quality-of-life burden), a cost per QALY threshold was estimated. This was subject to parameter and structural uncertainty, but a central estimate of £12,936 per QALY was reported [4].

There is growing recognition of the need for estimates of *k* that reflect opportunity costs in terms of health to inform resource allocation decisions in low-, middle-, and high-income countries [10], [11]. Nevertheless, with the exception of the work by Claxton et al., there is a lack of empirically based estimates of *k*. This article draws out the implications of what the limited available evidence suggests about supply-side CETs (*k*s) in a range of jurisdictions. We return to the subject of how these estimates might be used to inform resource allocation decisions in the Discussion section.

## Methods

The estimate of *k* by Claxton et al. [4] is based on the estimates of the marginal productivity of health care spending in just one jurisdiction. In principle, a similar approach could be adopted to estimate the relationship between health care spending and health outcomes internationally, using countries as units of analysis, to determine *k* in a wide range of settings.

To date, however, cross-country evidence on the productivity of health care spending has focused on answering the question “Does health care spending improve health outcomes?” Recent research adjusting for potential reverse causality in this relationship (e.g., the possibility of governments spending more when health outcomes are worse) suggests that the answer to this question is *yes* [12]. The available literature, however, does not focus on how the effect of health care spending on health outcomes varies according to the level of health care spending or country income. The available analyses do suggest that the marginal productivity of health care spending diminishes with increasing health care spending or country income [13], [14], [15]. This indicates that the threshold varies with country income or health care spending and reflects our expectation that the amount of health displaced by new resource commitments decreases as country income or health care spending rises. There is, however, little information to quantify how the marginal productivity of health care spending varies with country income.

There is a body of literature that estimates *v* (the consumption value of health or the demand-side threshold) in different countries. Some of this literature is based on stated-preference elicitation of individuals’ WTP for morbidity-adjusted life-years (e.g., QALYs) [16], [17], but a larger body of work estimates the “value of a statistical life” (VSL) by estimating individuals’ WTP for mortality reductions (e.g., by estimating wage compensation for on-the-job risk exposure) [18], [19]. Moreover, this literature also examines how the VSL varies across jurisdictions as a function of national per capita income (i.e., the elasticity of the VSL with respect to income, *ε*). This potentially provides information about the income elasticity of *v* if we can assume that the income elasticity of the VSL is equal to the income elasticity of the value of a life-year, and this, in turn, is equal to the income elasticity of the value of a morbidity-adjusted life-year (e.g., QALY). For this to be the case across countries, a VSL must convert to the same number of QALYs across countries.

Understanding the income elasticity of *v* across countries raises an interesting prospect. If a similar income elasticity of *k* exists as for *v*, income elasticities of the VSL can be applied to the Claxton et al. estimate of *k* for the English NHS to provide estimates of *k* in a wide range of jurisdictions. For the income elasticity of *k* to equal that of *v* requires the ratio between *k* and *v* to. We follow this approach to provide estimates of *k* for application in different countries on the basis of their per capita income levels, the CET for the English NHS, the per capita income in the United Kingdom, and the elasticity of VSL with respect to income.

**Fig. 1:**
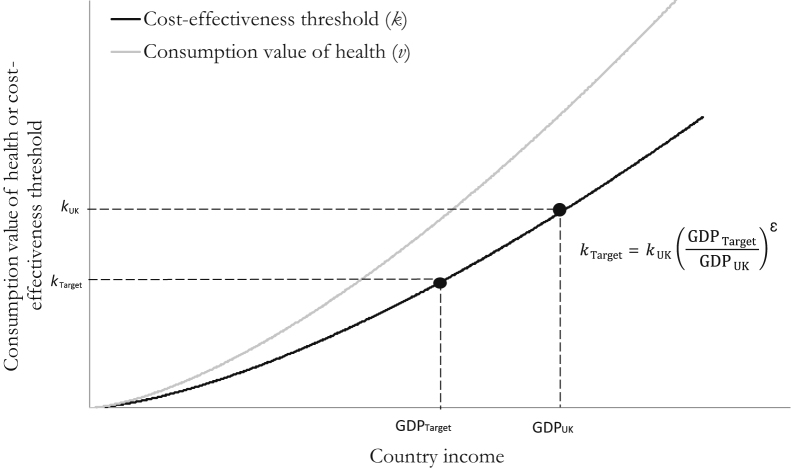
Method for inferring country-specific cost-effectiveness thresholds from the UK threshold.

This approach is illustrated in Figure 1 and, in summary, requires the following three assumptions: 1) that the relative discrepancy between *v* and *k* is constant across countries; 2) that the values used for *k* and *ε* are appropriate estimates; and 3) that the income elasticity of the VSL equals the income elasticity of the consumption value of a QALY. These assumptions are examined in the Discussion section. Nevertheless, we note that the broad expectation that both *v* and *k* will increase with country income is uncontentious. As income increases, basic consumption needs are met and individuals become more willing to exchange income for health (*v*), and health care spending expands accordingly. As income and health care expenditure rise, the marginal productivity of health care spending diminishes (*k* increases). Our model requires that health care spending increases such that the predicted increase in *k* is observed. We, however, make no assumptions regarding how the expansion to health care is funded. It could be funded via an expansion to the tax base, a redistribution of the tax base, or a combination of the two.

The best available estimate of the UK CET is £12,936 per QALY ($18,609 purchasing power parity [PPP]-adjusted [20]). The GDP per capita estimates for 2013 were obtained from the World Bank data set. In line with the literature on the VSL, elasticities were applied to countries’ GDP per capita adjusted for PPP [21] (see, e.g., Milligan et al. [22]). CETs are reported in 2013 PPP-adjusted US dollar values. Values without PPP adjustment are also provided alongside non–PPP-adjusted GDP [23]. We use data on the ratio of the PPP conversion factor to the market exchange rate to remove the PPP adjustment but retain the presentation in US dollars [24].

### Estimates of Income Elasticity for Demand for Health

The relationship between the VSL and per capita income at the level of jurisdictions is investigated in a small but emerging literature [19], [22], [25]. The literature has evolved out of a longer standing body of work that has examined the relationship between income and health valuation at the level of individuals (i.e., “within” countries) [18], [25]. Of central interest in both these bodies of work (i.e., within-group, at the individual level, and between-group, at the level of jurisdictions) is the income elasticity of the value of health.

Initial empirical research conducted primarily in higher income countries among individuals, and most often in the United States, suggested elasticities in the range of 0.4 to 0.6 [18], [19]. These estimates came mainly from cross-sectional studies looking at wage-risk premiums. The estimates, however, have been described as “nonsensical” when extrapolated to lower income countries because the corresponding VSL would be beyond the ranges considered plausible [19].

The methods to estimate the income elasticities of VSL have, therefore, been more carefully scrutinized in more recent years. In particular, cross-sectional (within-group) estimates from earlier studies have been contrasted with longitudinal or cohort (between-group) studies (which typically estimate elasticities >1 even within countries) and reasons for inconsistencies have been explored [19], [25], [26]. For instance, Aldy and Smyth [26] used a life-cycle model applied to US data on the consumption and labor supply choices faced by individuals with uncertain life expectancy and wage income to explain this discrepancy. They argued that cross-sectional studies are more likely to capture changes in realized income, whereas longitudinal or across cohort studies capture the impact of permanent income (i.e., reflecting lifetime opportunities to generate income), which is more informative when translating VSL estimates across countries. Estimates of elasticity with respect to realized income are lower because realized income is more variable.

The recent consensus then is that the income elasticity of VSL to transfer estimates across countries should be more than 1 [19], [22]. A range of elasticities were selected for this analysis (1.0, 1.5, and 2.0) to reflect uncertainty in the literature. On the basis of the study by Milligan et al. [22], a function of an elasticity of 0.7 was also applied for high-income countries (those with GDP per capita >$10,725, 2005 price year, PPP), and of 2.5 for countries with per capita incomes less than this threshold. In line with the recommendations by Milligan et al., the elasticities from this study were applied to 2013 PPP-adjusted GDP, deflated to reflect 2005 international dollars. The resulting threshold values were then inflated to reflect 2013 international dollars.

## Results

**Fig. 2:**
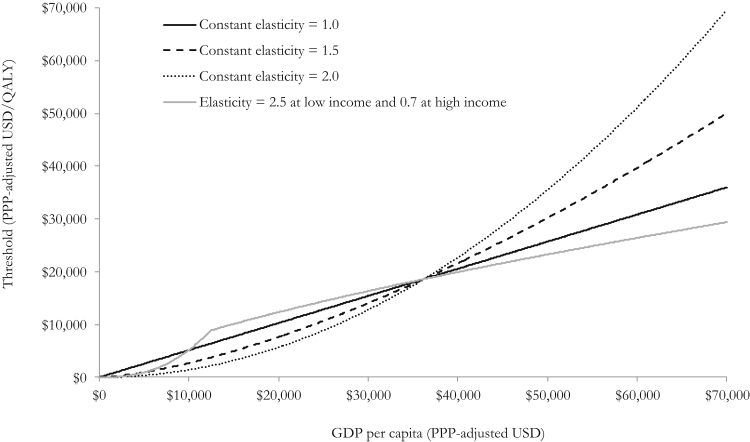
Predicted cost-effectiveness threshold (*k*) values by country income. GDP, gross domestic product; PPP, purchasing power parity; QALY, quality-adjusted life-year; USD, US dollar.

Predicted CETs across country income levels are shown in Figure 2 for a range of income elasticities for the VSL. Higher income elasticities imply lower CETs in countries with lower GDP per capita and higher CETs in countries with higher GDP per capita compared with the United Kingdom. The impact of alternative choices of elasticity is larger because the discrepancy between the GDP of the country of interest and the UK GDP widens. Results for a selection of specific countries are presented in Table 1.

US dollar CET values with and without PPP adjustment are provided in the Supplemental Materials found at http://doi:10.1016/10.1016/j.jval.2016.02.017 for all countries for which data were available from the World Bank database for 2013. Values without adjustment for PPP can be converted to local currency using standard exchange rates.

As exemplar countries, for Malawi (the country with the lowest per capita income in the world), Cambodia (with borderline low and low-middle income), El Salvador (with borderline low-middle and upper-middle income), and Kazakhstan (with borderline high-middle and high income), CETs were estimated to be $3 to $116 (1%–51% GDP per capita), $44 to $518 (4%–51% GDP per capita), $422 to $1967 (11%–51% GDP per capita), and $4485 to $8018 (33%–59% GDP per capita), respectively. For Luxembourg (the country with the highest per capita income in the world), we estimated a CET of $43,092 to $143,342 (39%–129% GDP per capita).

## Discussion

Policymakers in all countries, whether classified as high-, middle-, or low-income countries, face difficult decisions about how to allocate scarce health care resources. CEA offers a means by which to compare the costs and health gains from interventions as a basis to inform investment decisions. For the results of CEA to align with population health improvement, health gains from recommended interventions must exceed the health foregone when resources are committed to those interventions. CETs should therefore reflect our best estimates of the opportunity cost of health care spending (*k*) and not the consumption value of health (*v*).

In this article we provided indicative estimates of CETs on the basis of opportunity costs (the *k*s) in a number of countries that are intended to reflect the likely marginal productivity of their health care systems. Because of the lack of attention paid to estimating *k* in the literature to date, the estimates are based on limited data and strong, uncertain assumptions. The estimated CETs are substantially lower than those used at present by decision-making agencies and international organizations. Compared with a threshold of $50,000 per QALY that has been conventionally applied in the United States [5], our approach estimated a CET in the range $24,283 to $40,112 per QALY. Even more starkly, the thresholds we estimated were far less than the 1 to 3 times GDP per capita suggested by the WHO for use in low- and middle-income countries [7], [8]. In the country with the lowest income in the world, Malawi, we estimated a CET of $3 to $116 (1%–51% GDP per capita), and in Kazakhstan, a country on the borderline between being a middle- and a high-income country, we estimated a CET of $4485 to $8018 (33%–59% GDP per capita). This implies that resource allocation decisions based on WHO thresholds are likely to be recommending interventions that can lead to reductions in population health.

A separate question is how the estimates of *k* should be used by health care decision makers. It is argued here that understanding the full net health effects of an intervention is essential. Decision makers must understand not only the magnitude of direct health gains from an intervention but also the health that is expected to be displaced by the intervention costs. An understanding of what the health effects of increasing or reducing health expenditure are likely to be (a supply-side threshold, *k*) is therefore necessary if social and political choices regarding resource allocation are to be made in an informed and accountable way. It is clear, therefore, that in estimating the full net health effects of an intervention, only those costs that fall on the health care budget should be included. This approach should, however, never be considered as a single decision-making rule, and instead should be an input into a wider decision-making process that is likely to include a range of additional considerations including important social value judgments and appropriate consideration of the effects decisions are likely to have outside of health (e.g., impact on financial protection). Nevertheless, understanding the opportunity cost on health of using these additional considerations is important to guide decisions. Indeed, in the context of a financially constrained health care system, any widening of the measure of benefit that informs decisions should also be reflected in terms of opportunity costs (e.g., to what extent will the financial protection benefits of alternative interventions be foregone).

Estimates of the consumption value of health (*v*) have no role in decisions regarding the allocation of the scarce available resources for delivering health care [1]. Estimates of the consumption value of health may have a role in informing the social choice of what level of resources should be devoted to health care. Nevertheless, estimates of individuals’ willingness to trade off personal consumption for the collective health gains of increased health care spending might be more useful for this. It is not clear whether any study to date has estimated this quantity.

The results presented rely on some core assumptions if they are to provide reasonable estimates of the marginal productivity of other (non-UK) health care systems. The plausibility of each assumption is considered in turn.

1. That the discrepancy between the consumption value of health (*v*) and the CET for health (*k*) is constant in relative terms across countries:

It is assumed that the proportionate “underfunding” of health care, through collectively pooled resources relative to individual preferences over consumption and health, is constant across countries.

The ratio between *k* and *v* is in fact likely to differ across country income levels. The most obvious reason for this is that health care budgets may differ across countries for reasons other than differences in valuations of health. In lower income countries, the size of the health care budget is likely to be constrained by the ability of countries to raise tax revenues. The difficulties faced by low-income countries in raising tax are well documented and include the presence of a large informal sector, the impact of aid on the size of the state, poor checks and balances that reduce the likelihood of common-interest spending, interest groups reducing the propensity to tax, low support for higher taxation due to perceptions of corruption, and the availability of institutions to facilitate tax collection [27]. This is likely to create a downward pressure on the health care budget (not reflected in our analysis) that will cause *k* to be lower than we predict. As well as having implications for *k*, the reduced size of the health care system may result in a greater demand for private health care spending. There may also be constraints on the ability of countries to allocate the available tax base to health as opposed to other state-funded programs.

In lower income countries, and particularly for the poorest countries, donor funding may represent a significant component of health care spending. The available evidence supports some substitution of donor funding for government health spending, although the substitution is partial [28]. The net effect of donor funding is, therefore, expected to increase public health care spending and therefore raise *k*.

Depending on whether the restrictions on health care expenditure and the influx of donor funding increase or decrease the budget beyond what it would otherwise be, *k* may be smaller or larger than predicted by this analysis.

2. That the UK estimate of *k* is correct and that the range of income elasticities (1–2) explored includes the correct value:

A recent systematic review [29] identified additional studies estimating the impact of health expenditure on health outcomes. Two of these studies were by Martin et al. [30], [31] and were precursors to the Claxton et al. work [4]. Lichtenberg [32] developed a production function for mortality reductions using US data. In this model, health is generated by using health care expenditures of previous years and the stock of medical innovations. The methods used, however, do not allow us to disentangle the impact of time trends in expenditure from other temporal influences on health and, therefore, are unlikely to provide a robust estimate of *k*.

The UK estimate of *k* is firmly founded on empirical estimation of the effect of changes in expenditure on mortality outcomes while accounting for endogeneity. The assumptions and judgment required are summarized in Table 32 of the study by Claxton et al. [33], which also provides links to text and footnotes in which the qualitative effect of these assumptions are examined in greater detail. The analysis made use of the best available existing evidence and, if anything, is more likely to be conservative than optimistic with respect to the health effects of changes in NHS expenditure, that is, is more likely to have overestimated rather than underestimated the UK threshold [34].

A range of values for the elasticity of the VSL (*ε*) was considered, informed by the literature; it should, however, be noted that there is little robust data on what value *ε* should take. Furthermore, expressions of WTP for individual health gains may differ markedly from individuals’ willingness to trade consumption for collective health gains, further increasing the uncertainty around the estimates used.

3. That the income elasticity of WTP for a QALY can be approximated by *ε*:

For the income elasticity of the VSL to equal the income elasticity of the WTP for a QALY, a statistical life saved should provide the same units of morbidity-adjusted health (e.g., QALYs) across countries. This could be questioned if lives saved were expected to generate very different remaining morbidity-adjusted life expectancies. Although life expectancy at birth varies considerably across countries, remaining life expectancy differs much less because of differences in age demographic characteristics. For example, Hammitt and Robinson [19] find remaining life expectancies of between 34 and 45 years in countries with widely varying per capita incomes. This is a result of much older populations in countries with higher life expectancies at birth. Although quality of life is likely to increase with income, older populations would also be expected to have higher levels of morbidity, and so differences in QALYs may also be small.

Therefore, although our results are embedded with many assumptions, it is not immediately clear whether these are likely to lead to our estimates of *k* being positively or negatively biased.

These results should be regarded as only a first attempt to inform this area of crucial policy importance. Further empirical evidence is required to inform decision makers’ understanding of *k*.

Although correlations between health care expenditure and health are well established, estimates of the *causal* effect of expenditure on health are few. Analysis of cross-country data could be used to inform international estimates of the marginal productivity of health care spending, and to estimate the income elasticity of *k* and estimate *k* for different countries, reflecting their demographic characteristics, epidemiology, health expenditure, income, and other covariates [35].

Within-country research could take a number of forms. When data are available, the analysis of Claxton et al. could be repeated. Econometric analyses of policy reforms and other natural experiments could also inform estimates of the marginal productivity of health spending. Another approach could be to explore the cost-effectiveness of interventions that are at present provided within a country and those falling outside of the budget envelope. In this way, policymakers can undergo a process of “threshold seeking” [36] and become more informed about *k* as the number of CEAs in their jurisdiction increases. One example of a study using an approach similar to this is from Malawi, which suggests a threshold of no more (and perhaps less) than $150 in that country [37], which is within the range ($3–$116) estimated here. Countries could also examine specific disinvestment opportunities to understand the health likely to be displaced by new investments. Similarly, where spending is made up of a relatively small number of interventions, a mathematical programming approach may be feasible [38], [39]. This approach identifies the optimal set of interventions to adopt from a given budget. The ICER of the least cost-effective funded intervention provides an estimate of the CET.

Most importantly, any research intended to inform CETs should focus on estimating the opportunity cost of health care spending, that is, should focus on *k* and not *v*. As more empirical evidence emerges for specific countries, there may also be value in synthesizing this information to provide better-informed extrapolations across countries.

## Conclusions

To date there have been no estimates of opportunity-cost-based CETs (*k*s) for low- or middle-income countries. This article draws out the implications of the limited available evidence to estimate opportunity-cost–based CETs for a range of countries. The overall conclusion is that the balance of evidence suggests that CETs used to date—such as the WHO estimates—are too high and should not be used to inform resource allocation decisions. Further research is needed to inform this key but neglected question. In the meantime, decision makers may want to use estimates generated here alongside country-specific information on the opportunity cost of health care funds to inform their resource allocation decisions.

## Figures and Tables

**Table 1:** Example results for a range of countries and the World Bank income classification cutoffs (2013 GDP per capita)

Country/income classification	PPP-adjusted (2013 US $)		Actual values (2013 US $)		Threshold as % GDP per capita
	GDP per capita	Threshold range*	GDP per capita	Threshold range*	
Country					
Malawi	780	9–401	226	3–116	1–51
Indonesia	9,559	1,298–4,914	3,475	472–1,786	14–51
Chile	21,911	6,819–13,141	15,732	4,896–9,436	31–60
Kazakhstan	23,206	7,648–13,675	13,610	4,485–8,018	33–59
United Kingdom	36,197	18,609–18,609	41,787	20,223–20,223	48–48†
Canada	43,247	21,051–26,564	51,958	25,292–31,915	49–61
United States	53,143	24,283–40,112	53,042	24,283–40,112	46–75
Norway	65,461	28,057–60,862	100,819	43,211–93,736	43–93
Income classification					
Low/middle income‡	1,045	16–537	NA	NA	1–51
Middle/high income‡	12,746	2,307–9,028	NA	NA	18–71

GDP, gross domestic product; NA, not available/applicable; PPP, purchasing power parity.

⁎ Reflects range of values obtained when using elasticity estimates of 1.0, 1.5, 2.0, and 2.5 for GDP less than $10,725 (2005 PPP US $) and 0.7 for GDP greater than $10,725 (2005 PPP US $).

† For the United Kingdom, the World Bank ratio of PPP conversion factor to market exchange rate did not correspond to the ratio of reported actual GDP to reported PPP-adjusted GDP. The threshold as a % GDP value for the United Kingdom, therefore, depends on whether PPP-adjusted or actual data are used (51% and 48%, respectively).

‡ We have assumed gross national income per capita to be the same as PPP-adjusted GDP. These values relate to the income cutoffs for low- to middle-income and middle- to high-income countries as defined by the World Bank.
