# Supplementary material for: Country-Level Cost-Effectiveness Thresholds: Initial Estimates and the Need for Further Research
Source: Value Health. 2016 Dec;19(8):929–35. doi: 10.1016/j.jval.2016.02.017 (PMC5193154; doi:10.1016/j.jval.2016.02.017)
Supplement: Supplementary file 1 — Supplementary material [file mmc1.docx]

**Appendix: All country values**

The table below presents the PPP-adjusted and non-adjusted range of threshold values for each country for which PPP-adjusted GDP was reported in the World Bank database. In some cases data was not available to remove the PPP-adjustment and only PPP-adjusted threshold values are reported.

| **Country** | **Cost-effectiveness threshold range** | |
| --- | --- | --- |
|  | **(USD, PPP adjusted)** | **(actual USD)** |
| Afghanistan | 56 - 1,023 | 19 - 349 |
| Albania | 1,563 - 5,816 | 702 - 2,612 |
| Algeria | 2,514 - 9,300 | 1,012 - 3,743 |
| Angola | 807 - 3,875 | 603 - 2,897 |
| Antigua and Barbuda | 6,250 - 12,750 | 3,965 - 8,090 |
| Armenia | 858 - 3,997 | 387 - 1,801 |
| Australia | 21,153 - 26,938 | 32,771 - 41,732 |
| Austria | 21,355 - 27,684 | 23,727 - 30,759 |
| Azerbaijan | 4,172 - 11,085 | 1,901 - 5,051 |
| Bahrain | 21,245 - 27,277 | 11,962 - 15,358 |
| Bangladesh | 93 - 1,315 | 30 - 427 |
| Belarus | 4,407 - 11,297 | 1,895 - 4,857 |
| Belgium | 20,060 - 23,111 | 22,570 - 26,003 |
| Belize | 1,012 - 4,340 | 584 - 2,503 |
| Benin | 46 - 921 | 20 - 414 |
| Bhutan | 835 - 3,943 | 267 - 1,258 |
| Bolivia | 534 - 3,151 | 250 - 1,474 |
| Bosnia and Herzegovina | 1,318 - 4,952 | 644 - 2,421 |
| Botswana | 3,490 - 10,419 | 1,621 - 4,839 |
| Brazil | 3,210 - 10,122 | 2,393 - 7,544 |
| Brunei Darussalam | 29,901 - 73,137 | 16,065 - 39,294 |
| Bulgaria | 3,609 - 10,541 | 1,720 - 5,025 |
| Burkina Faso | 38 - 840 | 17 - 379 |
| Burundi | 8 - 396 | 3 - 137 |
| Cabo Verde | 584 - 3,297 | 343 - 1,935 |
| Cambodia | 131 - 1,564 | 44 - 518 |
| Cameroon | 104 - 1,394 | 49 - 654 |
| Canada | 21,051 - 26,564 | 25,292 - 31,915 |
| Central African Republic | 5 - 310 | 3 - 171 |
| Chad | 61 - 1,070 | 31 - 540 |
| Chile | 6,819 - 13,141 | 4,896 - 9,436 |
| China | 2,013 - 7,957 | 1,151 - 4,550 |
| Colombia | 2,174 - 8,754 | 1,370 - 5,518 |
| Comoros | 35 - 801 | 19 - 452 |
| Congo, Dem. Rep. | 8 - 384 | 5 - 230 |
| Congo, Rep. | 489 - 3,016 | 264 - 1,628 |
| Costa Rica | 2,733 - 9,574 | 2,006 - 7,027 |
| Cote d'Ivoire | 129 - 1,548 | 61 - 737 |
| Croatia | 6,206 - 12,720 | 3,953 - 8,101 |
| Cyprus | 12,318 - 16,130 | 11,020 - 14,430 |
| Czech Republic | 10,620 - 15,322 | 7,325 - 10,569 |
| Denmark | 20,888 - 25,974 | 28,767 - 35,771 |
| Djibouti | 128 - 1,541 | 71 - 857 |
| Dominica | 1,429 - 5,205 | 991 - 3,611 |
| Dominican Republic | 1,943 - 7,618 | 937 - 3,675 |
| Ecuador | 1,557 - 5,788 | 858 - 3,191 |
| Egypt, Arab Rep. | 1,745 - 6,669 | 522 - 1,993 |
| El Salvador | 856 - 3,991 | 422 - 1,967 |
| Equatorial Guinea | 16,150 - 17,717 | 9,843 - 10,798 |
| Eritrea | 20 - 615 | 9 - 280 |
| Estonia | 8,912 - 14,418 | 6,574 - 10,636 |
| Ethiopia | 26 - 696 | 10 - 255 |
| Fiji | 897 - 4,086 | 507 - 2,307 |
| Finland | 19,334 - 20,781 | 23,867 - 25,653 |
| France | 18,861 - 19,347 | 21,168 - 21,713 |
| Gabon | 5,268 - 12,018 | 3,164 - 7,218 |
| Gambia, The | 39 - 857 | 12 - 252 |
| Georgia | 729 - 3,683 | 366 - 1,850 |
| Germany | 21,080 - 26,668 | 21,933 - 27,747 |
| Ghana | 224 - 2,043 | 104 - 951 |
| Greece | 9,345 - 14,658 | 7,982 - 12,520 |
| Grenada | 1,878 - 7,302 | 1,272 - 4,948 |
| Guatemala | 756 - 3,750 | 360 - 1,788 |
| Guinea | 22 - 645 | 9 - 269 |
| Guinea-Bissau | 22 - 639 | 9 - 256 |
| Guyana | 610 - 3,368 | 348 - 1,924 |
| Haiti | 41 - 875 | 20 - 421 |
| Honduras | 299 - 2,360 | 149 - 1,177 |
| Hong Kong SAR, China | 24,302 - 40,202 | 17,409 - 28,801 |
| Hungary | 7,434 - 13,540 | 4,268 - 7,773 |
| Iceland | 19,942 - 22,720 | 22,567 - 25,712 |
| India | 416 - 2,781 | 115 - 770 |
| Indonesia | 1,298 - 4,914 | 472 - 1,786 |
| Iran, Islamic Rep. | 3,450 - 10,378 | 1,054 - 3,171 |
| Iraq | 3,276 - 10,194 | 1,504 - 4,679 |
| Ireland | 21,071 - 26,634 | 23,063 - 29,153 |
| Israel | 15,243 - 17,366 | 16,821 - 19,163 |
| Italy | 16,712 - 17,928 | 16,867 - 18,094 |
| Jamaica | 1,122 - 4,570 | 668 - 2,719 |
| Japan | 18,651 - 18,731 | 19,769 - 19,854 |
| Jordan | 1,971 - 7,757 | 872 - 3,432 |
| Kazakhstan | 7,648 - 13,675 | 4,485 - 8,018 |
| Kenya | 73 - 1,164 | 32 - 519 |
| Kiribati | 49 - 954 | 43 - 848 |
| Korea, Rep. | 15,598 - 17,505 | 12,227 - 13,722 |
| Kosovo | 1,085 - 4,493 | 473 - 1,961 |
| Kyrgyz Republic | 147 - 1,651 | 58 - 649 |
| Lao PDR | 329 - 2,474 | 113 - 852 |
| Latvia | 7,532 - 13,602 | 5,133 - 9,270 |
| Lebanon | 4,187 - 11,098 | 2,420 - 6,416 |
| Lesotho | 95 - 1,329 | 41 - 581 |
| Liberia | 11 - 451 | 6 - 234 |
| Libya | 6,503 - 12,927 | 3,697 - 7,349 |
| Lithuania | 9,175 - 14,565 | 5,598 - 8,886 |
| Luxembourg | 35,195 - 117,072 | 43,092 - 143,342 |
| Macao SAR, China | 48,116 - 288,671 | 30,832 - 184,977 |
| Macedonia, FYR | 1,978 - 7,791 | 824 - 3,246 |
| Madagascar | 28 - 717 | 9 - 235 |
| Malawi | 9 - 401 | 3 - 116 |
| Malaysia | 7,709 - 13,712 | 3,481 - 6,192 |
| Maldives | 1,929 - 7,550 | 1,103 - 4,318 |
| Mali | 38 - 844 | 17 - 368 |
| Malta | 12,965 - 16,419 | 10,138 - 12,838 |
| Marshall Islands | 196 - 1,908 | 182 - 1,774 |
| Mauritania | 131 - 1,564 | 46 - 550 |
| Mauritius | 4,202 - 11,112 | 2,248 - 5,945 |
| Mexico | 3,850 - 10,780 | 2,410 - 6,749 |
| Micronesia, Fed. Sts. | 180 - 1,829 | 162 - 1,646 |
| Moldova | 310 - 2,400 | 148 - 1,151 |
| Mongolia | 1,264 - 4,849 | 543 - 2,085 |
| Montenegro | 2,912 - 9,786 | 1,464 - 4,921 |
| Morocco | 736 - 3,702 | 316 - 1,590 |
| Mozambique | 16 - 537 | 8 - 294 |
| Namibia | 1,332 - 4,979 | 791 - 2,958 |
| Nepal | 72 - 1,154 | 22 - 357 |
| Netherlands | 21,104 - 26,757 | 23,153 - 29,354 |
| New Zealand | 17,226 - 18,117 | 20,555 - 21,619 |
| Nicaragua | 297 - 2,350 | 118 - 937 |
| Niger | 12 - 469 | 5 - 213 |
| Nigeria | 446 - 2,880 | 239 - 1,545 |
| Norway | 28,057 - 60,862 | 43,211 - 93,736 |
| Oman | 21,322 - 27,562 |  |
| Pakistan | 314 - 2,416 | 87 - 669 |
| Palau | 3,235 - 10,149 | 2,531 - 7,940 |
| Panama | 5,352 - 12,083 | 3,042 - 6,869 |
| Papua New Guinea | 92 - 1,305 | 75 - 1,073 |
| Paraguay | 919 - 4,135 | 484 - 2,179 |
| Peru | 1,969 - 7,747 | 1,114 - 4,383 |
| Philippines | 606 - 3,358 | 256 - 1,421 |
| Poland | 7,694 - 13,703 | 4,440 - 7,908 |
| Portugal | 9,527 - 14,756 | 7,738 - 11,985 |
| Puerto Rico | 17,145 - 18,088 | 14,075 - 14,849 |
| Qatar | 45,558 - 246,565 | 31,105 - 168,345 |
| Romania | 4,932 - 11,746 | 2,467 - 5,875 |
| Russian Federation | 8,263 - 14,046 | 5,007 - 8,511 |
| Rwanda | 30 - 746 | 13 - 323 |
| Samoa | 363 - 2,598 | 265 - 1,897 |
| Sao Tome and Principe | 125 - 1,527 | 68 - 827 |
| Saudi Arabia | 24,484 - 41,080 | 11,799 - 19,797 |
| Senegal | 73 - 1,166 | 34 - 544 |
| Serbia | 2,175 - 8,760 | 1,061 - 4,275 |
| Seychelles | 8,310 - 14,074 | 5,470 - 9,265 |
| Sierra Leone | 53 - 990 | 23 - 435 |
| Singapore | 31,889 - 88,068 | 22,342 - 61,701 |
| Slovak Republic | 9,686 - 14,841 | 6,561 - 10,053 |
| Slovenia | 11,374 - 15,690 | 9,135 - 12,603 |
| Solomon Islands | 61 - 1,063 | 57 - 1,004 |
| South Africa | 2,221 - 8,909 | 1,175 - 4,714 |
| South Sudan | 77 - 1,198 | 40 - 617 |
| Spain | 14,638 - 17,124 | 13,277 - 15,531 |
| Sri Lanka | 1,346 - 5,005 | 453 - 1,686 |
| St. Kitts and Nevis | 6,222 - 12,731 | 4,110 - 8,409 |
| St. Lucia | 1,584 - 5,914 | 1,107 - 4,133 |
| St. Vincent and the Grenadines | 1,615 - 6,058 | 998 - 3,746 |
| Sudan | 162 - 1,734 | 84 - 901 |
| Suriname | 3,740 - 10,672 | 2,286 - 6,525 |
| Swaziland | 634 - 3,436 | 288 - 1,559 |
| Sweden | 21,148 - 26,917 | 28,306 - 36,028 |
| Switzerland | 24,450 - 40,914 | 36,661 - 61,348 |
| Tajikistan | 90 - 1,291 | 37 - 533 |
| Tanzania | 45 - 912 | 18 - 357 |
| Thailand | 2,941 - 9,820 | 1,181 - 3,943 |
| Timor-Leste | 71 - 1,153 |  |
| Togo | 27 - 715 | 13 - 327 |
| Tonga | 399 - 2,726 | 333 - 2,275 |
| Trinidad and Tobago | 13,159 - 16,503 | 7,941 - 9,959 |
| Tunisia | 1,747 - 6,680 | 678 - 2,592 |
| Turkey | 5,114 - 11,895 | 2,950 - 6,861 |
| Turkmenistan | 2,784 - 9,635 | 1,588 - 5,495 |
| Tuvalu | 188 - 1,870 | 200 - 1,991 |
| Uganda | 28 - 725 | 11 - 293 |
| Ukraine | 1,097 - 4,518 | 487 - 2,005 |
| United Kingdom | 18,609 - 18,609 | 20,223 - 20,223 |
| United States | 24,283 - 40,112 | 24,283 - 40,112 |
| Uruguay | 5,450 - 12,160 | 4,548 - 10,147 |
| Uzbekistan | 379 - 2,656 | 138 - 965 |
| Vanuatu | 127 - 1,538 | 139 - 1,685 |
| Venezuela, RB | 4,701 - 11,553 | 3,724 - 9,151 |
| Vietnam | 398 - 2,721 | 144 - 982 |
| Yemen, Rep. | 223 - 2,035 | 83 - 757 |
| Zambia | 144 - 1,635 | 68 - 768 |
| Zimbabwe | 41 - 874 | 21 - 455 |
